# Effects of Dietary *Melissa officinalis* Supplementation on Hematological, Neuroendocrine, and Behavioral Parameters in Weaned Piglets and in the Beginning of the Fattening Period

**DOI:** 10.3390/life16091539

**Published:** 2026-09-15

**Authors:** Mariyana Petrova, Petya Veleva, Sonya Ivanova

**Affiliations:** 1 Agricultural Institute—Shumen, 9700 Shumen, Bulgaria; mari_anna1305@abv.bg; 2 Agricultural Academy, 1373 Sofia, Bulgaria; 3 Faculty of Digital and Green Technologies, Trakia University, 6000 Stara Zagora, Bulgaria; petya.veleva@trakia-uni.bg

**Keywords:** weaned pigs, welfare, *Melissa officinalis*, serotonin, cortisol, behaviour, agonistic interactions, social hierarchy

## Abstract

This study aimed to evaluate the effects of dietary supplementation with *Melissa officinalis* on the haematological, biochemical, neuroendocrine, and behavioural parameters of weaned pigs, as well as on behaviours associated with the establishment of social hierarchy following transfer to the fattening stage under conditions of social competition. The study included 48 pigs allocated to control and experimental treatments. Over the 55-day experimental period, pigs in the experimental treatment received dried *Melissa officinalis* leaf material incorporated into the diet, with the daily amount gradually increasing from 3.20 to 9.60 g/animal/day. The mean daily amount administered throughout the experimental period was 6.99 g/animal/day, approximately 7.0 g/animal/day. During the growing period, behavioural responses, serum serotonin and cortisol concentrations, and haematological and biochemical parameters were assessed. Following transfer to the fattening stage, agonistic behaviour associated with the establishment of social hierarchy was monitored. The data were analysed using one-way analysis of variance and correlation analysis. Dietary supplementation with *Melissa officinalis* significantly increased white blood cell (WBC; *p* = 0.048), lymphocyte (LYM; *p* = 0.008), and monocyte counts (MON; *p* = 0.004), as well as serum serotonin concentrations (*p* < 0.001). Serum cortisol concentrations were 37.73% lower in the experimental treatment than in the control treatment, although the difference was not statistically significant (*p* = 0.304). Pigs in the experimental treatment exhibited lower locomotor activity (*p* = 0.001), a tendency towards less climbing behaviour (*p* = 0.059), and a longer duration of play fighting (*p* = 0.001). Following transfer to the fattening phase, pigs in the experimental treatment exhibited a lower frequency of agonistic interactions during the establishment of social hierarchy (*p* = 0.008). In the control treatment, cortisol was negatively correlated with active behaviour (r = −0.845; *p* < 0.01) and positively correlated with inactive behaviour (r = 0.845; *p* < 0.01), whereas serotonin was positively correlated with play fighting (r = 0.462; *p* < 0.05). These findings indicate that the inclusion of dried *Melissa officinalis* leaf material in the diet improves immune status, beneficially modulates neuroendocrine regulation, and facilitates the social adaptation of pigs by reducing agonistic behaviour. Therefore, *Melissa officinalis* may represent a promising phytogenic feed additive for mitigating social stress and improving pig welfare during critical production stages.

## 1. Introduction

Council Directive 2008/120/EC (Council of the European Union, 2009), which lays down minimum standards for the protection of pigs, and Council Directive 98/58/EC (Council of the European Union, 1998), concerning the protection of animals kept for farming purposes, place particular emphasis on safeguarding pig welfare. Measures aimed at limiting social aggression and its adverse consequences focus primarily on controlling and reducing conflict-related interactions among animals [1], which are characteristic of modern intensive pig production systems. Current production cycles require pigs to be housed in different facilities and to undergo repeated regrouping and transfer before being sent to slaughter. Weaning is the first of these stressful procedures and occurs at a relatively early age. Piglets are simultaneously separated from the sow, mixed with unfamiliar conspecifics, placed in a novel environment, and transitioned to solid feed lacking the antibodies and protective factors provided by maternal milk. This combination of stressors activates the hypothalamic–pituitary–adrenocortical (HPA) axis and consequently induces an acute increase in cortisol concentrations, irrespective of weaning age, indicating both physiological and psychological stress [2]. Moreover, relocation disrupts the pre-existing social hierarchy and elicits post-mixing aggression, which serves to re-establish social order among unfamiliar pigs [3,4]. Similar behaviour is also observed when pigs are transferred to the fattening stage. Following regrouping, aggressive interactions may persist for up to 28 days and, under conditions of intense competition for feed, for as long as 56 days [5]. Reducing stress in pig production is therefore essential not only for improving animal welfare but also for optimising zootechnical and productive performance [6].

Accordingly, effective and sustainable approaches are required to mitigate stress and improve behavioural responses in pigs. Phytogenic feed additives are increasingly recognised as a promising strategy in livestock nutrition because of their broad range of biological effects, including antioxidant and anti-stress activities [7]. In this context, *Melissa officinalis* represents a promising phytogenic additive for pigs exposed to social and physiological stress. *Melissa officinalis* (lemon balm), a member of the family *Lamiaceae*, has been extensively investigated because of its neuroprotective, antioxidant, and anxiolytic properties [8,9]. Its biological activity is primarily attributed to its phenolic compounds, including rosmarinic and caffeic acids, and flavonoids such as quercetin, kaempferol, and apigenin [10,11]. These compounds exert potent antioxidant and anti-inflammatory effects and contribute to maintaining central nervous system homeostasis, thereby providing protection against neurodegenerative and behavioural disorders [12,13,14]. By modulating neurotransmitter systems and reducing neuronal excitability, dietary administration of the plant in various forms may help reduce aggressive behaviour and improve the overall physiological welfare of animals [15,16]. Pharmacological evidence indicates that *Melissa officinalis* interacts with several neurotransmitter systems, including the GABAergic, serotonergic, and cholinergic systems [17].

Despite the established biological and neuromodulatory properties of *Melissa officinalis*, information regarding its use as a feed additive in pigs, particularly its potential to modulate responses to social stress, remains limited. Previous studies have focused primarily on the plant’s pharmacological, antioxidant, and neuroprotective properties, whereas the effects of its dietary inclusion on the relationship between neuroendocrine parameters and behavioural responses in weaned pigs remain insufficiently understood. In particular, little is known about whether dietary supplementation with *M. officinalis* can influence subsequent behavioural adaptation and agonistic interactions when animals are regrouped at the beginning of the fattening period. Therefore, the novelty of the present study and its aim is an integrated assessment of the effects of *M. officinalis* as a feed additive on interrelated behavioural, neuroendocrine, haematological, and biochemical parameters in weaned pigs, together with the evaluation of their behavioural responses to subsequent regrouping and the establishment of a new social hierarchy under conditions of social competition.

## 2. Materials and Methods

### 2.1. Experimental Design in the Weaned Pig Feeding and Melissa officinalis Supplementation

A 55-day experimental trial involving weaned pigs of the Danube White breed was conducted at the Agricultural Institute—Shumen. The pigs were weaned at 40 days of age, with a mean initial body weight of 9.233 kg. By the end of the trial, the pigs had reached a mean body weight of 25.552 kg. The animals were randomly allocated to two treatments—a control treatment (Treatment I) and an experimental treatment (Treatment II)—with equal representation of both sexes. Each treatment comprised 24 animals (12 males and 12 females) distributed among four pens of six pigs each. Thus, a total of 48 pigs were housed in eight pens, as shown in Table 1.

The pigs were fed a standard diet formulated for weaned pigs, the composition of which is presented in Table 2. Pigs in the experimental treatment received dried *Melissa officinalis* leaf material (Herbal Pharmacy–Herbs Komers, Sofia, Bulgaria), previously ground into a fine powder. The supplement was manually incorporated and thoroughly homogenised into the standard diet according to a predefined supplementation schedule (Table 3). Supplementation in the experimental treatment commenced immediately after weaning, on Day 1 of the experimental period.

The dried *M. officinalis* leaf material was introduced gradually, beginning at 3.20 g/pig/day on Day 1 of the trial. The daily amount was progressively increased to a maximum of 9.60 g/pig/day during the experimental period to facilitate the adaptation of the newly weaned pigs to the phytogenic supplement. The calculated mean daily amount of dried *M. officinalis* leaf material administered over the entire 55-day experimental period was 6.95 g/pig/day, considered approximately 7.0 g/pig/day in the present study.

The pigs were fed using tube feeders and provided with water through nipple drinkers. The indoor microclimate was regulated by a Big Dutchman computer-controlled system (Big Dutchman International GmbH, Vechta, Germany) in accordance with the husbandry and veterinary requirements for weaned pigs.

### 2.2. Blood Sampling and Laboratory Analyses

At the end of the experimental period, blood samples were collected from 12 pigs per treatment from the orbital venous sinus using a closed blood collection system. All samples from the control and experimental treatments were collected between 08:00 and 09:00 h from fasted animals before the morning feeding. The pigs were routinely fed within a standardised time interval between 08:00 and 08:30 h; however, on the day of blood collection, feeding was performed after sampling had been completed. Blood samples were collected into plastic blood collection tubes (Vacusera, Izmir, Turkey), which were immediately inverted 10 times. Samples intended for serum biochemistry were collected in serum tubes and allowed to clot at room temperature for 2–3 h before centrifugation at 2000× *g* for 15 min, using a BOECO SC-8 centrifuge (Boeckel + Co. GmbH + Co. KG, Hamburg, Germany). The serum was separated and stored at −20 °C until subsequent biochemical analysis. Whole-blood samples were collected in EDTA-containing tubes and maintained at room temperature for haematological analysis within 6 h of collection.

Haematological analyses were performed at the Agricultural Institute–Shumen using an Exigo H400 automated veterinary haematology analyser (Boule Diagnostics AB, Spånga, Sweden), in accordance with the manufacturer’s instructions. Cortisol and serotonin analyses were conducted by Ramus, an external accredited laboratory. Serum cortisol concentrations were determined by chemiluminescent immunoassay (CLIA) using an automated Abbott Alinity i immunoassay analyser (Abbott Laboratories, Abbott Park, IL, USA) and the Cortisol Reagent Kit (Abbott, 08P33), with an analytical sensitivity of 0.7 µg/dL.

Serum serotonin concentrations were determined by liquid chromatography–tandem mass spectrometry (LC–MS/MS) using a Vanquish Flex UHPLC system coupled to a TSQ Quantis triple-quadrupole mass spectrometer (Thermo Fisher Scientific, Waltham, MA, USA). The analysis was performed using the Chromsystems IVD method for serotonin determination (Chromsystems Instruments & Chemicals GmbH, Gräfelfing, Germany), with a limit of detection (LOD) of <3 µg/L and a limit of quantification (LOQ) of 10 µg/L.

### 2.3. Video Surveillance in the Weaned Pigs

A DAHUA IPC-F42P video camera (Zhejiang Dahua Technology Co., Ltd., Hangzhou, China; 4 MP; resolution 1920 × 1080; infrared night vision; fixed wide-angle lens) was mounted on the ceiling above each of the four pens and connected to a DH-XVR1B04 recording device (Zhejiang Dahua Technology Co., Ltd., Hangzhou, China), an XVR supporting multichannel recording and H.264 compression. The cameras were positioned to provide complete visual coverage of all areas within the monitored pens, irrespective of ambient light conditions. The behaviour of the piglets in each treatment was continuously video-recorded for 24 h/day on two consecutive days at both the beginning and the end of the experimental period. Following a preliminary review of the 24 h recordings, the interval from 08:00 to 18:00 h was selected for quantitative behavioural analysis because, under the specific housing conditions, the pigs’ locomotor, social, and play activities occurred predominantly during daylight hours. All behavioural data were obtained from video footage digitally recorded on Days 1 and 2 following weaning and mixing, as well as on the two days preceding the end of the experiment (Days 53 and 54).

Active and inactive behaviours were assessed using instantaneous scan sampling at 2 min intervals, resulting in 600 observations per group (Table 4). Locomotor–rotational play and social play behaviours were monitored continuously, and their durations were recorded in seconds (Table 5). Behaviour was scored directly by a single trained observer using predefined behavioural categories and recording criteria. The same observer analysed all video recordings to ensure consistent application of the behavioural criteria and to eliminate inter-observer variability.

### 2.4. Experimental Design in the Fattening Category—Video Surveillance

A total of 40 pigs from the control treatment (Treatment I) and the experimental treatment (Treatment II) were transferred to the fattening stage at a mean body weight of 25.552 kg. The pigs were equally allocated to two pens, with 20 animals per pen. They were fed a standard grower diet formulated for fattening pigs (Table 6) using tube feeders, while water was provided through nipple drinkers. The indoor microclimate was regulated by a Big Dutchman computer-controlled system.

A DAHUA IPC-F42P video camera (Zhejiang Dahua Technology Co., Ltd., Hang-zhou, China; 4 MP; resolution 1920 × 1080; infrared night vision; fixed wide-angle lens) was mounted on the ceiling above each of the two pens and connected to a DH-XVR1B04 recording device (Zhejiang Dahua Technology Co., Ltd., Hangzhou, China), an XVR sup-porting multichannel recording and H.264 compression. The cameras were positioned to provide complete visual coverage of all areas within the monitored pens, irrespective of ambient light conditions. The behaviour of the pigs in each treatment was continuously video-recorded for 24 h/day on two consecutive days following their transfer and mixing at the beginning of the fattening period. All behavioural data were collected from the video recordings during the standardised interval from 08:00 to 18:00 h on Days 1 and 2 after transfer and mixing. The same daily observation interval as in the preceding behavioural assessments was used to standardise the evaluation and encompass the period of predominant behavioural and social activity. To assess the establishment of the social hierarchy within each pen, agonistic behaviour was monitored, and the duration of agonistic interactions was recorded in seconds (Table 7). Behavioural observations were performed by the same trained observer using the predefined behavioural categories and recording criteria.

### 2.5. Statistical Analysis

All statistical procedures were performed using IBM SPSS Statistics, version 26.0 (IBM Corp., Armonk, NY, USA). Normality of the data distribution was assessed using the D’Agostino–Pearson omnibus test, as well as the Shapiro–Wilk test for smaller samples. One Way ANOVA was applied to evaluate statistically significant differences between the control and experimental treatments in hematological parameters, hormonal levels (serotonin, cortisol), and behavioral parameters (LOC, SOC, agonistic behavior).

Correlation analysis was used to determine the relationships between hormonal parameters and specific behavioral responses. The analysis was performed using the Bivariate Pearson procedure to evaluate the relationships between hormonal parameters and the behavioral responses of the piglets. Correlation strength was interpreted as follows: 0 < |r| < 0.30 (weak); 0.30 < |r| < 0.50 (moderate); 0.50 < |r| < 0.70 (substantial); 0.70 < |r| < 0.90 (high); and 0.90 < |r| < 1.00 (very high).

## 3. Results

### 3.1. Hematological Profile

The haematological values obtained were interpreted by comparison with the published reference intervals for pigs provided by LABOKLIN (Laboratory for Clinical Diagnostics). The results presented in Table 8 indicate changes in several haematological parameters in growing pigs from the experimental treatment (Treatment II), which received an average of 7 g of dried *Melissa officinalis* leaf material per animal per day. Compared with the control treatment, the experimental treatment showed significantly higher white blood cell (WBC; *p* = 0.048), lymphocyte (LYM; *p* = 0.008), and monocyte (MON; *p* = 0.004) counts, although all values remained within the physiological reference ranges. A trend towards a higher relative proportion of lymphocytes (LYM%; *p* = 0.086) and a significantly lower relative proportion of granulocytes (GRA%; *p* = 0.048) were observed in pigs from the experimental treatment. The coefficients of determination (R^2^) for WBC, LYM, and MON were 0.166, 0.281, and 0.314, respectively, indicating that dietary treatment with *Melissa officinalis* accounted for 16.6%, 28.1%, and 31.4% of the observed variation in these parameters, respectively.

### 3.2. Serum Levels of Serotonin and Cortisol

**Figure 1:**
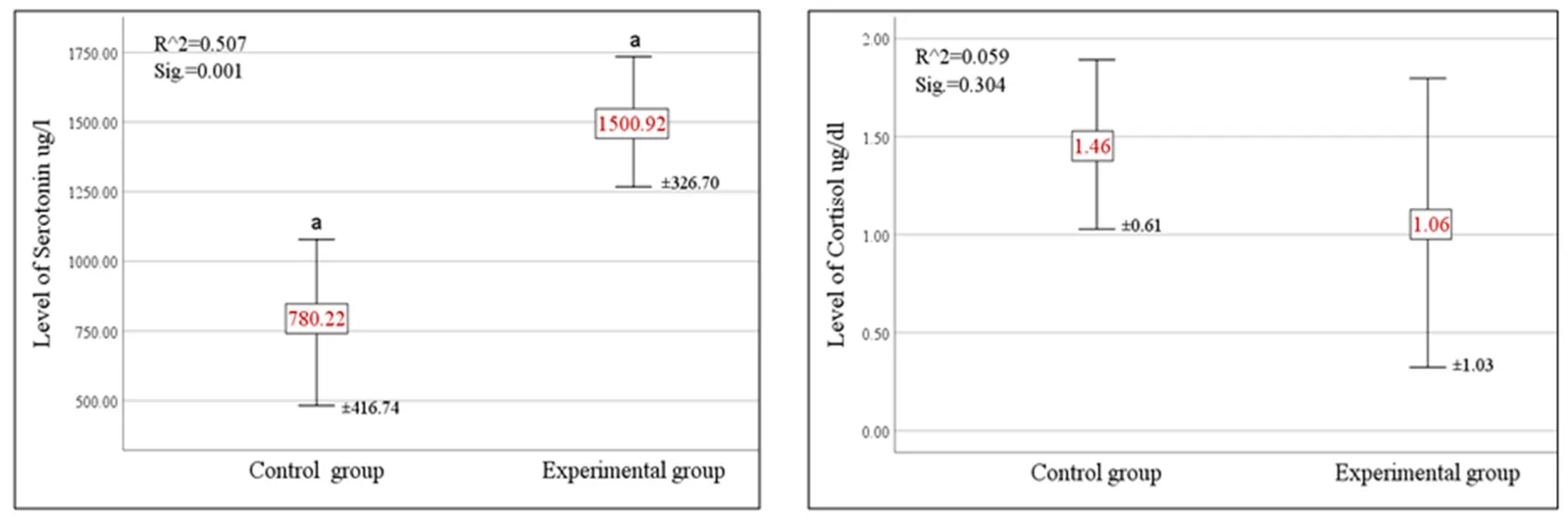
Serotonin and cortisol levels in control and experimental treatments of pigs supplemented with *Melissa officinalis*. Notes: Identical lowercase superscript letters indicate statistically significant differences between the control and experimental groups (*p* < 0.05).

Serum serotonin concentrations (Figure 1) were significantly higher (*p* < 0.001) in pigs receiving *Melissa officinalis*, whereas serum cortisol concentrations were 37.73% lower than those in the control treatment; however, the difference was not statistically significant (*p* = 0.304). The coefficient of determination (R^2^) indicated that dietary treatment with *Melissa officinalis* accounted for 50.7% (R^2^ = 0.507) of the observed variation in serum serotonin concentrations, whereas the corresponding proportion for cortisol was 5.9% (R^2^ = 0.059).

### 3.3. Behavioral Reactions

The data presented in Table 9 indicate that dietary supplementation with *Melissa officinalis* affected specific behavioural patterns included in the ethogram of locomotor–rotational play (LOC) and social play (SOC) in growing pigs. At the beginning of the experimental period, no statistically significant differences were observed between the control and experimental treatments in overall locomotor activity (*p* = 0.949), climbing (*p* = 0.108), or nudging behaviour (*p* = 0.316), indicating good baseline comparability between the treatments. At the end of the experimental period, pigs from the experimental treatment spent significantly more time inactive (lying) and, correspondingly, less time active (*p* = 0.001). In addition, pigs receiving *Melissa officinalis* tended to spend less time performing climbing behaviour (*p* = 0.059). Pigs from the experimental treatment exhibited a significantly longer duration of nudging (*p* = 0.031) and play-fighting behaviour (*p* = 0.001) than those from the control treatment. At the end of the experimental period, the coefficients of determination (R^2^) for nudging and play fighting were 0.141 and 0.199, respectively, indicating that dietary treatment with *Melissa officinalis* accounted for 14.1% and 19.9% of the observed variation in these behavioural parameters, respectively.

No statistically significant differences between the two treatments were observed at the end of the experimental period for the remaining recorded behavioural patterns, including pen exploration, scampering, pushing, social interactions between adjacent pens, and social interactions within the same pen (*p* > 0.05). Notably, social interactions within the same pen were the only behavioural measure that differed significantly between the treatments at the beginning of the experiment (*p* = 0.001). As this difference preceded dietary supplementation, it cannot be attributed to the effects of *Melissa officinalis*.

### 3.4. Agonistic Behavior in Forming the Social Hierarchy

**Figure 2:**
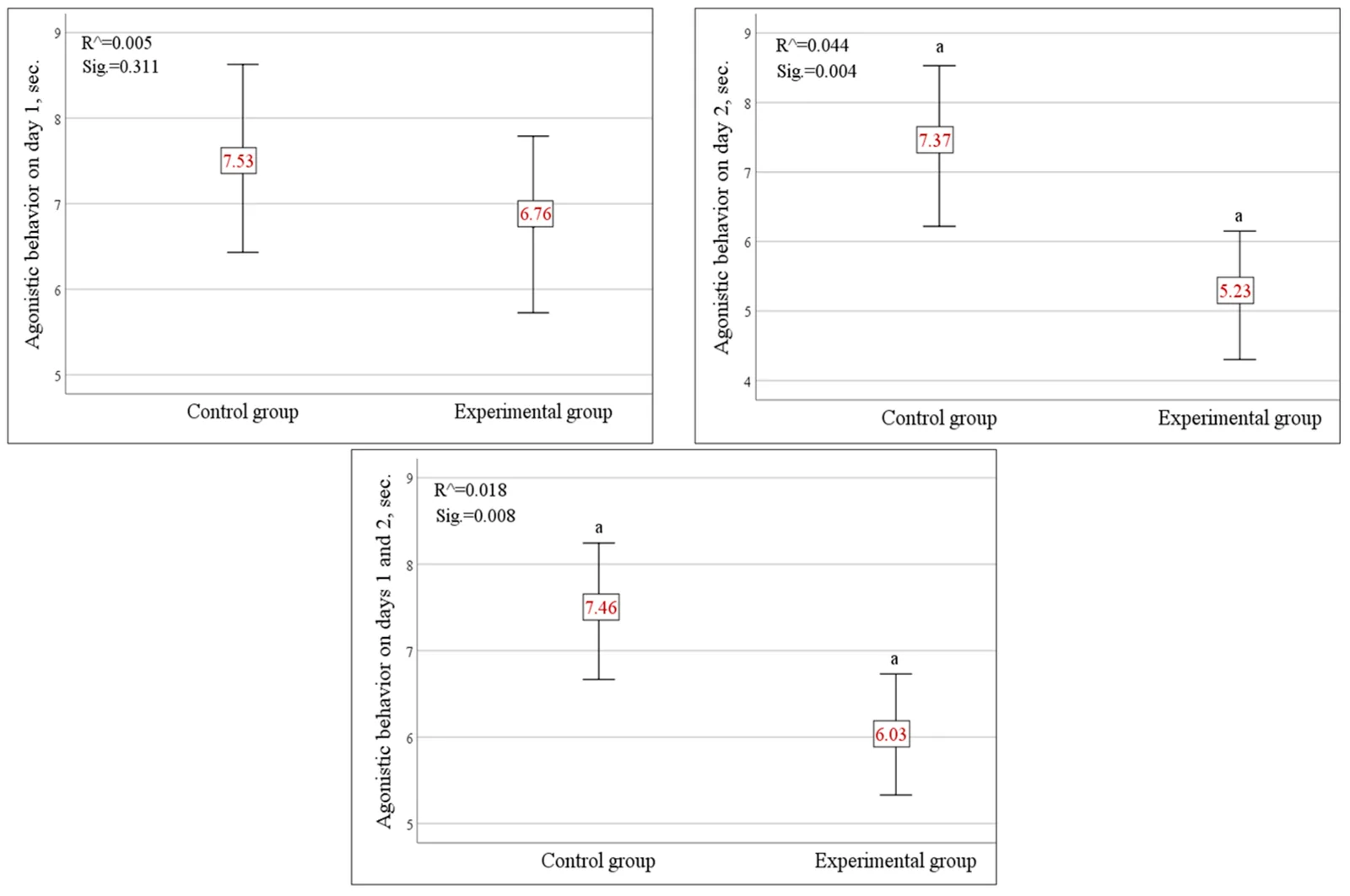
Agonistic behavior in control and experimental treatments of pigs supplemented with *Melissa officinalis*. Notes: Identical lowercase superscript letters indicate statistically significant differences between the control and experimental groups (*p* < 0.05).

The results presented in Figure 2 indicate that dietary supplementation with *Melissa officinalis* significantly affected the frequency of agonistic interactions during the establishment of social hierarchy. On the first day following group formation at the beginning of the fattening stage, no statistically significant difference in the frequency of agonistic interactions was observed between the control and experimental treatments (*p* = 0.311). On the second day, however, pigs receiving *Melissa officinalis* exhibited a significantly lower frequency of agonistic interactions than those in the control treatment (*p* = 0.004). The combined analysis of data from both observation days also demonstrated a significantly lower overall frequency of agonistic interactions in the experimental treatment (*p* = 0.008).

### 3.5. Correlations Between Neuroendocrine Indicators and Behavioral Responses

Table 10 presents the correlations between serum serotonin and cortisol concentrations and behavioural measures in the control and experimental treatments at the end of the experimental period. In the control treatment, serum cortisol concentrations were strongly negatively correlated with active behaviour (r = −0.845; *p* < 0.01) and strongly positively correlated with inactive behaviour (r = 0.845; *p* < 0.01). In addition, cortisol concentrations were moderately negatively correlated with nudging behaviour (r = −0.442; *p* < 0.05) and moderately positively correlated with the combined frequency of agonistic interactions recorded on Days 1 and 2 (r = 0.487; *p* < 0.05). Serum serotonin concentrations were moderately positively correlated only with play-fighting behaviour (r = 0.462; *p* < 0.05), whereas no statistically significant correlations were observed with the remaining behavioural measures (*p* > 0.05).

## 4. Discussion

### 4.1. Hematological and Neuroendocrine Effects of Melissa officinalis

Scientific evidence regarding the effects of *Melissa officinalis* on physiological parameters and behaviour in farm animals remains limited, and, to the best of our knowledge, no comprehensive studies in pigs have simultaneously evaluated its haematological, biochemical, and behavioural effects. The present study is the first to investigate the effects of dried *Melissa officinalis* leaf material in growing and fattening pigs through an integrated assessment of haematological parameters, neuroendocrine status (serotonin and cortisol), behavioural responses, and agonistic interactions during the establishment of social hierarchy following mixing. This comprehensive approach enables a more detailed understanding of the potential mechanisms through which *Melissa officinalis* modulates physiological adaptation, social behaviour, and the processes involved in establishing stable social relationships in pigs.

In the present study, weaned pigs receiving an average of 7 g of dried *Melissa officinalis* leaf material per animal per day exhibited significantly increased white blood cell (WBC), lymphocyte (LYM), and monocyte (MON) counts. Similar findings have been reported in other animal species. For example, Abou Zead et al. [26] found that broiler chickens fed diets containing 10 g of *Melissa officinalis*/kg of feed exhibited significant increases in leukocyte, lymphocyte, and monocyte counts (*p* < 0.0001). Comparable results were reported by Al-Gharawi et al. [27], who observed improvements in haematological and immunological parameters following the dietary inclusion of *Melissa officinalis* in broilers. According to Travel et al. [28], these changes reflect the immunomodulatory activity of the plant, which is associated with its high content of phenolic compounds, particularly rosmarinic acid, and other antioxidant constituents that mitigate oxidative and toxic stress. The fact that all haematological parameters in the present study remained within their physiological reference ranges suggests that the observed increases in WBC, LYM, and MON reflected enhanced immune responsiveness rather than the presence of an inflammatory process.

The biochemical findings further support the beneficial physiological effects of *Melissa officinalis*. In the present study, pigs from the experimental treatment exhibited significantly higher serum serotonin concentrations, whereas serum cortisol concentrations showed a non-significant decreasing trend. These findings suggest that *Melissa officinalis* may influence neuroendocrine regulation and adaptation to stress as well. *Melissa officinalis* has been shown to interact with several neurotransmitter systems, including the GABAergic, serotonergic, and cholinergic systems [13,17]. Rosmarinic acid, one of the principal bioactive constituents of the plant, inhibits GABA transaminase, thereby increasing the concentration of γ-aminobutyric acid (GABA) in the central nervous system and reducing neuronal excitability [29]. Moreover, rosmarinic acid has been shown to cross the blood–brain barrier and inhibit monoamine oxidase A (MAO-A) activity, thereby reducing serotonin degradation and increasing its availability in the brain [30]. Lin et al. [31] also reported that the administration of *Melissa officinalis* reduced serotonin turnover and increased its concentration in brain regions involved in emotional regulation. The combined modulation of GABAergic and serotonergic neurotransmission may therefore underlie the significant increase in serum serotonin concentrations observed in the present study, together with the associated changes in the animals’ behavioural responses.

Activation of the hypothalamic–pituitary–adrenocortical (HPA) axis is a key component of the physiological stress response and results in the release of glucocorticoids, primarily cortisol and corticosterone [32]. Several authors have reported that chronic stress increases corticosterone concentrations in laboratory animals [33,34,35]. In the present study, the tendency towards lower cortisol concentrations in pigs receiving *Melissa officinalis*, although not statistically significant, is consistent with published evidence regarding the anti-stress effects of the plant. Ghazizadeh et al. [30] observed reduced serum corticosterone concentrations in stress-exposed mice following the administration of a hydroalcoholic extract of *Melissa officinalis*, whereas Yoo et al. [36] reported a similar effect in mice treated with 50 or 200 mg/kg of the extract for three weeks. A comparable effect was described following the combined administration of *Melissa officinalis* and *Passiflora caerulea*, which resulted in significantly reduced corticosterone concentrations in mice [37]. Collectively, these findings suggest that *Melissa officinalis* supplementation may be associated with reduced serum cortisol concentrations and may therefore contribute to attenuation of physiological responses to stress. However, as only serum cortisol was measured, the present findings do not provide sufficient evidence to conclude that *Melissa officinalis* modulates HPA-axis activity as a whole.

### 4.2. Behavioral Effects of Melissa officinalis

The neurochemical changes were also reflected in the animals’ behaviour. In the present study, pigs receiving *Melissa officinalis* spent significantly more time inactive (lying) and less time engaged in locomotor activity. Concurrently, a tendency towards a shorter duration of climbing behaviour (Climb; photographic illustration) was observed. This behaviour was defined as placing the forelimbs on another pig or on elements of the pen equipment. Climbing is generally considered an expression of increased excitability, arousal, or social tension; therefore, its reduction in pigs receiving lemon balm may provide an additional indication of calmer behaviour and a lower level of arousal. These findings are consistent with evidence from experimental rat models showing that aqueous *Melissa officinalis* extract exerts dose-dependent anxiolytic and sedative effects. At a dose of 5 mg/kg, a pronounced anxiolytic effect was observed, whereas sedation predominated at higher doses, possibly through an opioid receptor-mediated mechanism [38]. Similar findings were reported by Ibarra et al. [39], who demonstrated that the chronic administration of *Melissa officinalis* extract reduced anxiety-like behaviour and altered circadian activity.

The characteristic aroma of *Melissa officinalis* should also be considered when interpreting the observed behavioural changes. Incorporating the dried leaf material into the diet altered its sensory characteristics and may therefore have influenced the animals’ responses to the feed and certain aspects of their behaviour. In the present study, the effect of odour was not evaluated independently and therefore cannot be distinguished from the other effects of the phytogenic supplement. Consequently, the possibility that the sensory properties of *M. officinalis* contributed to some of the observed behavioural changes cannot be entirely excluded. Future studies incorporating appropriate experimental controls would help distinguish effects associated with the sensory characteristics of the supplement from those attributable to its biologically active constituents.

Nudging behaviour (Nudge), the duration of which was significantly longer in pigs receiving *Melissa officinalis*, is also of particular interest. This behaviour was defined as gently touching another pig with the snout without direct snout-to-snout contact and was considered a social–exploratory rather than an aggressive interaction. A comparable increase in exploratory activity in laboratory animals was reported by Khabaeva et al. [40], who found that *Melissa officinalis*-based preparations exerted anxiolytic effects characterised by increased exploratory behaviour and reduced fear responses. Therefore, the longer duration of nudging observed in the present study may reflect lower anxiety and improved social adaptation.

### 4.3. Social Behavior and Hierarchy Formation in Growing Pigs

The findings related to agonistic behaviour are of particular practical relevance. In intensive pig production, mixing unfamiliar animals represents a major stressor because it necessitates the establishment of a new social hierarchy, which is accompanied by aggressive interactions [1,3,4]. In the present study, no differences were observed between the control and experimental treatments on the first day following the formation of the new groups, indicating a similar initial response to social stress. On the second day, however, the frequency of agonistic interactions was significantly lower in pigs receiving *Melissa officinalis*, and the same pattern was observed when data from both days were analysed jointly. This finding suggests a more rapid stabilisation of the social hierarchy and more successful adaptation to post-mixing conditions. The behavioural changes observed in the present study may also be considered in the context of the effects of other phytogenic additives used in pigs. Pastorelli et al. [41] found that dietary supplementation with *Passiflora incarnata* extract in weaned piglets was associated with less pronounced aggressive behaviour, increased exploratory activity, and lower cortisol concentrations compared with control animals. Similarly, Casal-Plana et al. [42] reported that supplementation with a phytogenic product containing *Valeriana officinalis* and *Passiflora incarnata* in growing pigs was associated with a tendency towards fewer negative social interactions and a lower number of skin lesions. These findings are consistent with the reduction in agonistic interactions observed in the present study in pigs receiving *M. officinalis*. Nevertheless, direct comparisons among different phytogenic additives should be made cautiously because of differences in plant species, supplement composition and formulation, dosage, duration of administration, and experimental conditions. With specific regard to *Melissa officinalis*, similar results were reported by Labalette et al. [43], who found that a dietary supplement containing *Melissa officinalis* extract combined with magnesium reduced the frequency of aggressive behaviour in growing pigs. In contrast to the present findings, Gazzola [44], who investigated a standardised dry extract of *Melissa officinalis* added to piglet feed from several days before weaning until 2–3 weeks thereafter to mitigate weaning stress, found no statistically significant effect on the number of skin lesions following mixing. These contrasting findings may be attributable to differences in the assessment methods employed. The number of skin lesions is an indirect measure of aggression, whereas direct behavioural observation using an ethogram enables the detection of dynamic social interactions and early behavioural changes.

### 4.4. Neuroendocrine–Behavioral Relationships

The correlation analysis further supports the association between neuroendocrine changes and pig behaviour. In both treatments, serum cortisol concentrations were strongly negatively correlated with active behaviour and positively correlated with inactive behaviour, supporting the role of cortisol as a sensitive physiological marker of adaptation to stressors. These relationships are consistent with the well-established function of the hypothalamic–pituitary–adrenocortical axis in regulating behavioural responses to stress.

More notable differences were observed for serotonin. In the control treatment, higher serum serotonin concentrations were associated with more pronounced play-fighting behaviour, whereas in pigs receiving *Melissa officinalis*, serotonin was negatively correlated with active behaviour, including nudging. At first glance, this finding may appear contradictory because the experimental treatment as a whole exhibited significantly longer durations of both nudging and play fighting. However, these findings are not mutually inconsistent. The between-treatment comparison demonstrates that *Melissa officinalis* supplementation increased the mean duration of these social behaviours, whereas the correlation analysis describes individual variation within the experimental treatment. Thus, pigs with the highest serum serotonin concentrations tended to exhibit lower overall locomotor activity, even though the experimental treatment as a whole displayed more social interaction.

These findings suggest that the effects of *Melissa officinalis* are not determined solely by increased serotonin concentrations but probably result from complex interactions among serotonergic, GABAergic, and neuroendocrine regulatory mechanisms. The plant is known to enhance GABAergic activity, thereby reducing neuronal excitability and anxiety, whereas the serotonergic system is involved in the regulation of social behaviour. Consequently, the animals may exhibit lower overall locomotor activity while simultaneously engaging in more calm, non-aggressive social interactions.

The magnitude of the treatment effect, expressed by R^2^, was particularly informative for the individual behavioural measures. The highest value was observed for play fighting (R^2^ = 0.199), followed by nudging (R^2^ = 0.141), whereas the effect on overall locomotor activity was considerably smaller (R^2^ = 0.039). These results indicate that *Melissa officinalis* exerted a greater influence on the quality of social interactions than on the overall level of locomotor activity. The increases in play fighting and nudging, combined with the concurrent reduction in agonistic interactions, suggest that the phytogenic additive shifted social behaviour towards non-aggressive forms of interaction. Play fighting in young pigs is regarded as a normal social behaviour that facilitates the development of social skills and the stabilisation of within-group relationships without causing injury. Therefore, the observed changes may reflect improved social adaptation, reduced anxiety, and the more rapid establishment of a stable social hierarchy in pigs receiving *Melissa officinalis*.

## 5. Limitations

The present study provides an integrated assessment of the effects of dried *Melissa officinalis* leaf material in pigs; however, the findings should be interpreted in light of several limitations. The number of animals included (48 pigs during the growing period and 40 pigs at the beginning of the fattening period) and the completion of the experiment within a single production cycle may have limited the statistical power and the generalisability of the findings to different production systems. The relatively short experimental follow-up period represents an additional limitation, as it did not allow the persistence of the observed effects during longer-term supplementation to be evaluated. Furthermore, the study included only Danube White pigs; therefore, the findings should be extrapolated to other breeds and genotypes with caution.

Another limitation is that all behavioural observations were performed by a single trained observer. Using the same observer throughout the analysis was intended to ensure consistent application of the predefined behavioural criteria and minimise inter-observer variability. Nevertheless, the absence of an independent second observer and a formal assessment of inter-observer reliability limited the independent evaluation of the reproducibility of the behavioural measurements. Future studies should therefore include multiple independent observers and an assessment of inter-observer reliability.

The present study evaluated haematological parameters and serum cortisol and serotonin concentrations as indicators related to the neuroendocrine response. However, a broader panel of additional biochemical and physiological markers associated with animal stress and welfare was not examined. This limited both a more comprehensive assessment of the physiological response to the supplement and the comparison of the observed behavioural changes with a wider range of physiological welfare indicators.

Future studies should include larger populations, different breeds or genotypes, alternative dosing regimens, longer follow-up periods, and a broader panel of biochemical and physiological markers associated with animal stress and welfare. Such an approach would enable a more comprehensive assessment of the effects of *Melissa officinalis* and help confirm its potential as a phytogenic feed additive for supporting welfare and social adaptation in pigs represents the first comprehensive investigation of the effects of dried *Melissa officinalis* leaf material in pigs; however, the findings should be interpreted in light of several limitations. The number of animals included (48 pigs during the growing period and 40 pigs at the beginning of the fattening period) and the completion of the experiment within a single production cycle may limit the statistical power of the study and the generalisability of its findings across different production systems. Future multicentre studies involving larger populations, different supplementation regimens, and longer follow-up periods are warranted. The inclusion of additional biomarkers of neuroendocrine regulation would also help confirm the potential of *Melissa officinalis* as a phytogenic feed additive for improving pig welfare and social adaptation.

## 6. Conclusions

In conclusion, the present study demonstrates that the dietary inclusion of dried *Melissa officinalis* leaf material in growing pigs beneficially affects neuroendocrine regulation and social behaviour during the establishment of social hierarchy. *Melissa officinalis* supplementation was associated with improved immunological parameters, increased serum serotonin concentrations, a tendency towards lower serum cortisol concentrations, reduced locomotor activity, and less pronounced agonistic behaviour, accompanied by more frequent non-aggressive social interactions. Collectively, these changes indicate improved adaptation to social stress and enhanced animal welfare following weaning.

## Figures and Tables

**Table 1:** Experiment scheme of the trial with addition of *Melissa officinalis* to the weaned pig diets.

Treatment	Control Treatment (I)	Experimental Treatment (II)
Number of animals	24	24
Dietary treatment	Basal diet	Basal diet + *Melissa officinalis* 7 g/head/day

Source: Authors’ own elaboration.

**Table 2:** Ingredient composition and calculated energy and nutrient content of the basal diet of weaned piglets.

Ingredient	%
Wheat	30.00
Barley	20.00
Maize	15.00
Bioconcentrate mixture-12 *	35.00
Total:	100.00
Calculated nutrient composition per kg of diet:	
Digestible energy, (kcal/kg)	3260
Metabolizable energy, (kcal/kg)	3150
Crude protein, (%)	18.00
Lysine, (g/kg)	8.35
Crude fiber, (%)	3.4
Crude fat, (%)	3.6
Nitrogen-free extract, (NFE, g/kg )	556
Calcium, (g/kg)	9.2
Phosphorus, (g/kg)	6.3

* Crude protein% 35.50; Crude fiber% 3.65; Crude fat% 6.59; Crude ash% 13.92; Vitamin A-45700 IU/kg; Vitamin D3 5900 IU/kg; Vitamin E 450 mg/kg; Iron total mg/kg 695.00; Copper total mg/kg 355.00; Lysine% 1.80; Methionine% 0.47; Threonine% 1.34; Calcium% 2.60; Sodium% 0.50; Phosphorus% 1.20; Zinc total mg/kg 390.00; Manganese total mg/kg 245.00; Selenium total mg/kg 1.45; Iodine total mg/kg 14.58.

**Table 3:** Daily administration schedule of *Melissa officinalis* during the 55-day experimental period.

Experimental Day	*Melissa officinalis* (g/head/day)
1–7	3.20
8–14	4.16
15–21	5.44
22–28	6.72
29–35	8.00
36–42	9.60
43–49	9.60
50–55	9.60

**Table 4:** Ethogram of active and inactive behaviors in domestic pigs (adapted from Statham et al., 2011 [18]; Rhim, 2012 [19]).

Behavior	Description
Inactive behavior (recorded by 2 min scan sampling)	Immobility or sleeping.
Active behavior (locomotion; recorded by 2 min scan sampling)	Any body movement, including feeding, drinking, walking, running, galloping, and rolling.

**Table 5:** Ethogram describing locomotor–rotational (LOC) and social play (SOC) behaviors in domestic pigs (adapted from Donaldson et al., 2002 [20]; Franchi et al., 2022 [21]).

LOC a* (locomotor–rotational play)	
**Behavior**	**Description**
Gambolling	Energetic galloping locomotion (both forelimbs moving almost simultaneously, followed by both hindlimbs) with forward bounding within the pen. Frequently accompanied by vigorous ear flapping, movement across a large area of the pen, and occasionally collisions with other pigs. During a gambolling bout, pigs may rapidly turn and change direction. The behavior ends when locomotion slows to walking or when the pig moves out of view.
Scamper	At least two consecutive forward bounds performed in rapid succession, usually accompanied by ear flapping and occasionally by head tossing. The behavior ends when locomotion slows or when the pig moves out of view.
Flop	A rapid transition from standing to sternal or lateral recumbency, appearing as a spontaneous collapse without physical contact with another pig.
Hop/Pivot	A short, bouncing jump in which all four feet leave the ground simultaneously; the body either remains oriented in the same direction or rotates rapidly around the vertical axis.
Turn	A rapid rotation of the body around its vertical axis while at least one foot remains in contact with the ground.
SOC b** (social play)	
**Behavior**	**Description**
Climb	Placement of both forefeet onto the back of another pig or onto the pen partition or feeder.
Socialization between pens	Attempts to establish contact with a pig housed in an adjacent pen through the pen partition.
Follow/Chase	Walking or running (often involving physical contact) in the same direction as another pig.
Lever	Lifting another pig with the snout so that at least two of its feet are raised off the floor.
Nudge	Gentle contact with another pig’s body using the snout, without snout-to-snout contact.
Push	Applying minimal to moderate force with the head, neck, or shoulders to push another pig’s body.
Play fight	Reciprocal head pushing and body contact between two pigs. Characterized by low-intensity fighting without biting or avoidance behavior, distinguishing it from agonistic fighting.
Within-pen social play	Non-aggressive social interactions between pigs within the same pen, including nudging, sniffing, licking, climbing, and playful physical contact, without signs of agonistic behavior.

a* Behavioral elements were considered mutually exclusive and were recorded with a clearly defined onset and termination. A pause in play lasting at least one second was considered the end of a play bout, and any subsequent play was recorded as a new event [20]. b** Energetic reciprocal interaction between two or more standing pigs, typically initiated as play fighting. During this behavior, pigs do not exhibit biting or avoidance responses. Social play ends when physical contact between the pigs ceases, when one pig loses interest, when chasing or following behavior stops, or when at least one of the pigs moves out of the observer’s field of view [21].

**Table 6:** Ingredient composition and calculated energy and nutrient content of the basal diet of fattening pigs.

Ingredient	%
Wheat	30.00
Barley	20.00
Maize	20.00
Bioconcentrate mixture-14 *	30.00
Total:	100.00
Calculated nutrient composition per kg of diet:	
Digestible energy, (kcal/kg)	3592
Metabolizable energy, (kcal/kg)	3308
Crude protein, (%)	17.94
Lysine, (g/kg)	0.90
Crude fiber, (%)	3.83
Crude fat, (%)	2.69
Nitrogen-free extract, (NFE, %)	70.39
Calcium, (g/kg)	0.85
Phosphorus, (g/kg)	0.68

* Crude protein% 35.80; Crude fiber% 8.12; Crude fat% 4.88; Crude ash% 14.37; Vitamin A-48700 IU/kg; Vitamin D3 6500 IU/kg; Vitamin E 277 mg/kg; Iron total mg/kg 710.00; Copper total mg/kg 320.00; Lysine% 1.33; Methionine% 0.79; Threonine–1.40; Calcium% 2.70; Sodium% 0.55; Phosphorus% 2.02; Zinc total mg/kg 375.00; Manganese total mg/kg 235.00; Selenium total mg/kg 1.34; Iodine total mg/kg 3.58.

**Table 7:** Ethogram of agonistic behavior [19,22,23,24,25].

Agonistic behavior	A dyadic encounter between two pigs involving physical contact (biting, head pushing, hitting, or shoving), in which the opponents are in an antiparallel position and both animals perform biting or striking actions. The encounter begins with the first physical contact and ends when one of the opponents displays submissive behavior (escape) or when the two piglets move away from each other.

**Table 8:** Mean hematological blood parameters in control and experimental treatments of pigs fed with *Melissa officinalis*.

Blood Parameters	Control Treatment (I), *n* = 12			Experimental Treatment (II), *n* = 12			*p*-Value	R^2^
	$\bar{x}$ ± SD	Min.	Max.	$\bar{x}$ ± SD	Min.	Max.		
**WBC G/L**	16.58 ± 6.88 ^a^	6.30	27.70	22.06 ± 5.89 ^a^	10.50	30.10	**0.048**	**0.166**
**LYM G/L**	5.12 ± 2.36 ^a^	2.20	8.80	7.99 ± 2.45 ^a^	3.20	11.60	**0.008**	**0.281**
**LY M%**	31.68 ± 7.00	14.40	37.70	35.92 ± 4.23	28.80	41.00	0.086	0.128
**MON G/L**	1.35 ± 0.59 ^a^	0.50	2.60	2.16 ± 0.65 ^a^	1.30	3.30	**0.004**	**0.314**
**MON%**	8.85 ± 2.37	4.90	12.00	10.11 ± 1.61	7.20	12.40	0.142	0.095
**GRA G/L**	10.12 ± 4.97	3.60	20.00	11.91 ± 3.15	6.00	16.40	0.303	0.048
**GRA%**	59.48 ± 8.28 ^a^	51.10	80.40	53.98 ± 3.82 ^a^	47.40	60.90	**0.048**	**0.166**
**HGB G/L**	151.50 ± 53.56	103.00	240.00	124.75 ± 40.50	91.00	231.00	0.181	0.080
**MCH/pg**	16.72 ± 0.74	15.30	17.80	16.74 ± 0.85	15.30	18.10	0.939	0.000
**MCHC G/L**	347.33 ± 22.63	322.00	385.00	336.92 ± 11.58	322.00	363.00	0.170	0.084
**RBC G/L**	9.00 ± 2.95	6.60	14.08	7.48 ± 2.46	5.24	13.45	0.184	0.079
**MCV/fl**	48.30 ± 3.08	44.20	52.70	49.78 ± 3.27	43.20	54.10	0.265	0.056
**HCT L/L**	0.43 ± 0.12	0.30	0.63	0.37 ± 0.11	0.28	0.65	0.182	0.080
**RDW/fl**	42.15 ± 3.89	37.50	52.30	41.35 ± 2.01	38.50	46.60	0.534	0.018
**RDW%**	19.30 ± 2.31	17.80	24.70	19.93 ± 2.04	16.60	23.00	0.490	0.022
**PLT G/L**	257.92 ± 130.47	104.00	436.00	335.92 ± 171.86	85.00	562.00	0.224	0.067
**MPV/fl**	7.67 ± 0.53	6.90	8.70	7.76 ± 0.50	6.80	8.20	0.668	0.009

Notes: **a**—Identical letters within a row indicate statistically significant differences between groups at *p* < 0.05; **SD**—Values are presented as mean ± standard deviation; **R^2^**—coefficient of determination. WBC—White blood cell count (G/L); LYM—Lymphocyte count (G/L); LYM%—Lymphocyte percentage (%); MON—Monocyte count (G/L); MON%—Monocyte percentage (%); GRA—Granulocyte count (G/L); GRA%—Granulocyte percentage (%); HGB—Hemoglobin concentration (g/L); MCH—Mean corpuscular hemoglobin (pg); MCHC—Mean corpuscular hemoglobin concentration (g/L); RBC—Red blood cell count (T/L); MCV—Mean corpuscular volume (fL); HCT—Hematocrit (L/L); RDW—Red cell distribution width, standard deviation (fL); RDW%—Red cell distribution width, coefficient of variation (%); PLT—Platelet count (G/L); MPV—Mean platelet volume (fL). We highlighted statistically significant results by bold formatting.

**Table 9:** Effect of *Melissa officinalis* supplementation on ethogram-based behavioral parameters in weaned pigs.

Behavioral Reactions	Control Treatment (I)	Experimental Treatment (II)	*p*-Value	R^2^
	$\bar{x}$ ± SD	$\bar{x}$		
Activity at the beginning, min	2.13 ± 2.28	2.12 ± 2.17	0.949	0.000
Inactivity at the beginning, min	3.88 ± 2.28	3.88 ± 2.17	0.978	0.000
Activity at the end, min	3.06 ± 2.29 ^a^	2.13 ± 2.33 ^a^	**0.001**	**0.039**
Inactivity at the end, min	2.94 ± 2.30 ^a^	3.87 ± 2.33 ^a^	**0.001**	**0.039**
Pen exploration at the beginning, sec	31.02 ± 14.53	32.71 ± 15.27	0.115	0.003
Pen exploration at the end, sec	29.54 ± 14.76	30.52 ± 16.087	0.451	0.001
Frenzied running at the beginning, sec	4.82 ± 3.179	6.06 ± 6.28	0.218	0.012
Frenzied running at the end, sec	5.56 ± 3.13	4.43 ± 2.70	0.461	0.039
Climbing at the beginning, sec	12.76 ± 8.07	15.55 ± 14.35	0.108	0.014
Climbing at the end, sec	20.14 ± 13.77	14.98 ± 10.05	0.059	0.046
Nudging at the beginning, sec	3.87 ± 3.14	3.34 ± 2.68	0.316	0.008
Nudging at the end, sec	2.15 ± 1.27 ^a^	5.00 ± 5.46 ^a^	**0.031**	**0.141**
Pushing at the beginning, sec	4.06 ± 3.34	3.71 ± 3.35	0.524	0.003
Pushing at the end, sec	2.57 ± 2.03	2.16 ± 1.33	0.275	0.014
Play fighting at the beginning, sec	9.14 ± 6.45	9.96 ± 7.372	0.403	0.003
Play fighting at the end, sec	8.11 ± 5.93 ^a^	16.22 ± 10.01 ^a^	**0.001**	**0.199**
Social interaction between pens at the beginning, sec	26.74 ± 13.52	29.23 ± 18.07	0.169	0.006
Social interaction between pens at the end, sec	27.53 ± 12.68	23.57 ± 13.49	0.168	0.021
Social interaction within a pen at the beginning, sec	25.73 ± 12.07 ^a^	21.18 ± 12.05 ^a^	**0.001**	**0.034**
Social interaction within a pen at the end, sec	28.21 ± 13.30	26.41 ± 14.29	0.531	0.004

Note: Values are presented as mean ± SD. Identical superscript letters within the same row indicate statistically significant differences between treatments (*p* < 0.05). SD—standard deviation; R—coefficient of determination. We highlighted statistically significant results by bold formatting.

**Table 10:** Correlation between hormone levels and pigs’ behavior at the end of the experimental period.

Behavioural Reactions	Active	Inactive	Nudging	Play Fighting	Agonistic Interaction on Day 2	Agonistic Interaction on Days 1 and 2
Control treatment						
Serotonin ug/L	−0.060	0.060	−0.114	0.462 *	−0.204	−0.220
Cortisol ug/dL	−0.845 **	0.845 **	−0.442 *	−0.194	−0.326	0.487 *
Experimental treatment						
Serotonin ug/L	−0.384 *	0.384 *	−0.593 *	−0.034	0.143	−0.003
Cortisol ug/dL	−0.758 *	0.758 *	−0.097	0.053	−0.082	−0.172

*—Correlation is significant at the 0.05 level (2-tailed) (in green). **—Correlation is significant at the 0.01 level (2-tailed) (in blue).

## Data Availability

The data supporting the findings of this study are available from the corresponding author upon reasonable request. The data are not publicly available because they form part of an ongoing research project and are being used in continuing analyses.
